# The Effects of Cattle Manure and Garlic Rotation on Soil under Continuous Cropping of Watermelon (*Citrullus lanatus* L.)

**DOI:** 10.1371/journal.pone.0156515

**Published:** 2016-06-03

**Authors:** Ruiping Yang, Yanling Mo, Changming Liu, Yongqi Wang, Jianxiang Ma, Yong Zhang, Hao Li, Xian Zhang

**Affiliations:** 1 College of Horticulture, Northwest A&F University, Yangling, China; 2 College of Biology Pharmacy and Food Engineering, Shangluo University, Shangluo, China; 3 Hanzhong City Agro-technology Extension Center, Hanzhong, China; Universita degli Studi di Pisa, ITALY

## Abstract

Continuous cropping of watermelon (*Citrullus lanatus* L.) can lead to reduced yield and quality. We aimed to determine the effects of cattle manure addition and rotation with green garlic to improve yield and reduce disease incidence in watermelon and to examine the effects on the biological and chemical characteristics of the soil. Field experiments were performed during 2012–2014 on land previously under two years of continuous watermelon cropping in northwest China. We examined three treatment combinations: watermelon and garlic rotation, cattle manure application before watermelon planting, and combined cattle manure addition and crop rotation. Watermelon monoculture was retained as a control. Watermelon yield was significantly higher and disease incidence was lower in the treatments than the control. The populations of soil bacteria and actinomycetes and the bacteria/fungi ratio increased significantly and soil enzyme activities were generally enhanced under treatments. Available nutrients and soil organic matter contents were much higher under experimental treatments than the control. Results suggest both cattle manure application and garlic rotation can ameliorate the negative effects of continuous cropping. The combined treatment of cattle manure addition and green garlic rotation was optimal to increase yield, reduce disease incidence and enhance soil quality.

## Introduction

Watermelon (*Citrullus lanatus* L.) is one of the most important summer fruits, and it is widely cultivated around the world. Watermelon production is a highly economically productive activity in China. However, watermelon crops, particularly under long-term continuous monoculture, can develop serious problems, such as low seed germination rates, high seedling mortality, stunted plant growth, leaf yellowing, morbidity, blight, leading to low fruit yield and quality [1]. The main causes of these problems with continuous watermelon cropping result from changes in the soil, particularly within the crop rhizosphere. Continuous cropping may alter the soil microbial community and produce soil abnormalities, such as deplete nutrients, erode the physical properties of the soil, and favor the accumulation of plant autotoxins [2]. Moreover, soil deterioration can have a negative feedback effect on the growth of the plants [3], which threatens the sustainable production of watermelon. Restoring soil health is critical to solving the problems associated with continuous watermelon cultivation.

Rotation of land out of watermelon cultivation is the oldest management practice to relieve continuous cropping problems. Previous studies have shown that crop yield and plant disease incidence can be affected by land management practices [4, 5]. Furthermore, soil enzyme activity, microbial population, and soil nutrient content were all found to be higher under crop rotation systems than under monoculture [6]. Garlic (*Allium sativum* L.), which is commonly used as a natural broad-spectrum antibiotic, may be effectively incorporated into a rotation system as a preceding crop [7]. Garlic has many benefits for use as a preceding crop; for example, garlic root exudates are an effective management measure against Phytophthora blight in pepper [8]. Abdel-Monaim and Abo-Elyousr found that intercropping with garlic was shown to decrease incidence of root rot fungi in lentil plants and produced the highest lentil seed yield in their study [9]. Studies have also shown that amendment with garlic essential oils or garlic straw can have an effective nematicidal or nematode inhibitory effect in soils under tomato crops [10, 10] and garlic straw addition can increase crop yields [10]. Furthermore, the garlic root exudates have been shown to produce noticeable effects on soil quality by altering soil pH, electrical conductivity, nutrient dynamics, and enzyme activity [12].

Recent interest in sustainable cropping systems has renewed attention on the use of organic materials as fertilizers. Livestock manures, which can enhance crop production and restore soil [13], are a preferred form of organic fertilizer. High organic carbon content can provide an instant energy source and boost soil microbial activity, and organic matter can improve poor soil physical conditions resulting from topsoil loss and compaction [14]. In addition, cattle manure can improve physical, chemical, and biological properties of soil [14], and is therefore an important option for maintaining sustainable agricultural practices.

The negative effects of soil erosion can be reversed by applications of cattle manure under crop rotation and this practice may provide long lasting effects [15]. Accordingly, successful management of continuous cropping problems may require a broad approach employing multiple changes in cultivation practices. Few studies have been conducted on the application of cattle manure [15] or crop rotation [4] for the control of problems associated with continuous cropping of watermelon. This paper reports the effects of cattle manure addition and rotation with green garlic on watermelon yield and Fusarium wilt *(Fusarium oxysporum f*. *sp*. *niveum)* susceptibility, as well as soil health as measured by biological and chemical properties. We aim to ascertain whether cattle manure addition and rotation with green garlic is effective for maintaining the soil quality under watermelon cropping, which will help ensure the sustainable long-term development of this crop.

## Materials and Methods

### Experimental design

The experiment was conducted from March 2010 to July 2014 under a plastic tunnel at the Xixiaozhai Experimental Station (N 34°17′, E 108°01′) of Northwest A&F University, Yangling, Shaanxi Province, China. Watermelons were cropped continuously for 5 years and only the final 3 years are tested in this study. Sampling began in the summer in June 2012 on land that had been under two years of continuous watermelon cropping. We used a brown loamy soil type for this experiment. The initial soil chemical properties were as follows: 70.83 mg·kg^−1^ available P, 206.32 mg·kg^−1^ available K, 39.37 mg·kg^−1^ available N, 18.64 g·kg^−1^ organic matter content, soil electrical conductivity (EC) of 441 mS·cm^−1^, and a soil pH of 7.84. The watermelon cultivar ‘Nongkeda 5’ used for the study is a crossbreed provided by the Watermelon and Melon Research Group at Northwest A&F University, Yangling, Shaanxi, China. Garlic (‘Caijiapo Hongpi’) and cattle manure were purchased in the local market (N 34°16′, E 108°04′). The cattle manure contained 104.38 mg·kg^−1^ available N, 46.63 mg·kg^−1^ available P, 178.64 mg kg^−1^ available K, 73.22 g·kg^−1^ organic material, EC of 292.31 mS·cm^−1^, and a pH of 8.84.

In this trial, we adopted a completely randomized design that spanned six growing seasons. The treatment design included two factors, green garlic rotation (G) and cattle manure (CM), and three experimental treatment combinations (listed in Table 1), including watermelon rotation with green garlic (W/G), application of cattle manure before watermelon planting (CM), and watermelon rotation with green garlic and application of cattle manure before planting (W/G+CM), as well as unamended watermelon monoculture as a control. Each treatment was replicated three times.

**Table 1:** Summary of the experimental design.

Treatment 1	Treatment 2	Treatment 3	Control
Garlic	Garlic	---	**---**
---	Cattle manure	Cattle manure	---
Watermelon	Watermelon	Watermelon	Watermelon

**Fig 1:**
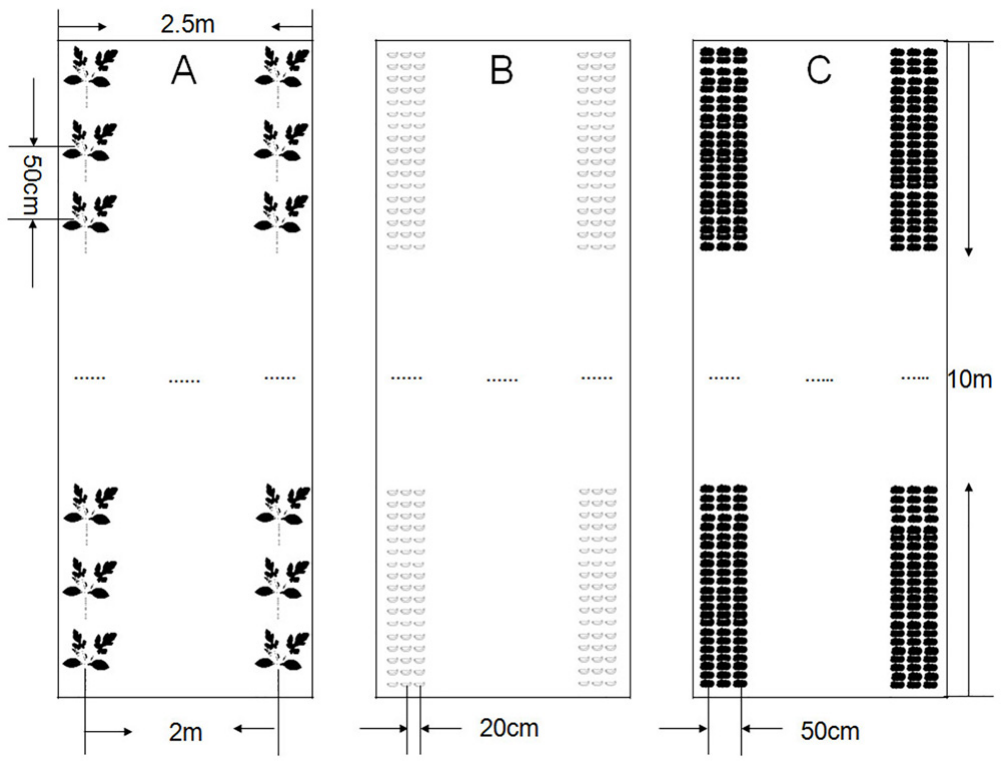
Watermelon planting and the treatment of garlic and cattle manure alone in the experiment. A: Density of watermelon planting; B: Density of garlic clove planting; C: Pattern of application of cattle manure.

The plastic tunnel was arranged in a north–south orientation, while plant lines were arranged in an east–west orientation. The watermelon planting lines adopted the model of high-ridged and plastic film cultivation, and drip irrigation under a plastic film. The crop rows were planted on ridges with a flat top that were 50 cm wide and 15 cm high (Fig 1A and 1C). There were three beds per plot for each of the three experimental treatments and the control treatment. Each bed was 10 m long and 2 m wide (Fig 1A and 1C). There were twenty plants per row, and it was 50 cm for plant spacing and 2 m for row spacing for different treatments (Fig 1A). Watermelon seedlings were planted on 31 March and harvested on June 15 in 2012, planted on 21 March and harvested on June 10 in 2013, and planted on March 26 and harvested on June 11 in 2014. Garlic cloves (20 cm row spacing and 8 cm plant spacing, with 375 cloves for each bed; approximately 0.8 T/ha; Fig 1B) were planted at the beginning of August and ploughed into the ground the following spring in the middle of February. Cattle manure was broadcasted into the bottoms of the planting rows (Fig 1C) at rates of 80 t·ha^−1^ (dry weight basis) before watermelon planting at the end of February or in early March.

### Plant yield and disease incidence

Watermelon fruits were harvested on June 15, 2012, on June 10, 2013, and on June 11, 2014. On each harvest, the total number of fruits and weights of fruits were measured. The number of seedlings infected by *Fusarium* wilt was assessed every day until 60 days after transplantation. Disease incidence was calculated according to the percentage of diseased plants out of the total number of plants growing in each block.

### Soil analysis

Soil adhering to the roots (at a depth of up to 20 cm) was gently dislodged each summer, one week after harvest. The soil samples were stored in insulated plastic bags and immediately transported to the laboratory. One portion of the soil sample was kept at 4°C to analyze the soil microbial population within three days, and the other portion of the sample was air-dried at room temperature (< 25°C) for measurement of biological and chemical properties. Triplicate samples were passed through a 1 mm sieve prior to analysis.

### Soil biological properties analysis

The culturable microbes were counted using a dilution and counting technique [16]. Briefly, 10 g soil was added to 90 mL sterile distilled water and shaken on a rotary shaker at 200 rpm for 30 min. Serial dilutions to 10^−9^ were prepared and 1 mL of each dilution was spread on beef extract-peptone medium to determine the bacterial population, on Martin’s Rose Bengal agar to determine the fungal population, and on Gaoshi No. 1 agar to determine the actinomycetes population. Soil enzyme activities were assayed based on the colorimetric determination using a spectrophotometer UV-6100 (Shanghai Meipuda instrument Co. LTD, Shanghai, China) of the product catalyzed by the enzymes. The pre-incubated soil subsamples were incubated with an appropriate substrate under standard conditions following the methods presented by Guan *et al*. [17]. Activities of urease, invertase, and alkaline phosphatase were measured using 10% urea, 8% sucrose, and 0.5% disodium phenyl phosphate, respectively, as substrates under standard conditions (24 h at 37°C). Protease and polyphenol oxidase activities were assayed using 1% casein and 1% pyrogallol as substrates under standard conditions for 24 h and 2 h at 30°C, respectively. Catalase activity was assayed using 0.3% hydrogen peroxide as a substrate after shaking for 20 min.

### Soil chemical properties analysis

Soil total phenolics were measured using Folin–Ciocalteu reagent [18]. They were assayed on the basis of the release and quantitative determination of the products of 4-hydroxybenzoic acid; soil samples were incubated with 4 mol L^−1^ sulfuric acid solution for 24 h at 110°C, and spectrophotometric measurements were then performed at 680 nm. The soil pH was measured in water with the soil: water ratio of 1:5 (*w*/*v*) [19] and the EC was measured at the same ratio [20]. Soil available N was assayed using a Kjeldahl analysis [21]. NH_4_^+^ was extracted with 2 M KCl and analyzed colorimetrically. Soil NO_3_^−^ was extracted with 0.5 M K_2_SO_4_ before colorimetric measurement at 220 nm and 270 nm. Available P was assayed using the methods of Sparks *et al*. [22]. Available K was estimated using an automated discrete analyzer CleverChem 200 (DeChem-Tech. GmbH, Hamburg, Germany) [22]. The organic matter content was assayed via the potassium dichromate volumetric method [23].

### Statistical analysis

Statistical analysis was conducted using the PASW Statistics 18.0 program (IBM; Armonk, NY, USA). All data were analyzed using a one-way analysis of variance, and differences between means were assessed using Duncan’s multiple range test (at a threshold of *P* < 0.05). The results are presented as the means ± standard deviation of 3–10 independent biological replicates.

## Results

### Effects of cattle manure and garlic rotation on watermelon yield and *Fusarium* wilt disease

**Fig 2:**
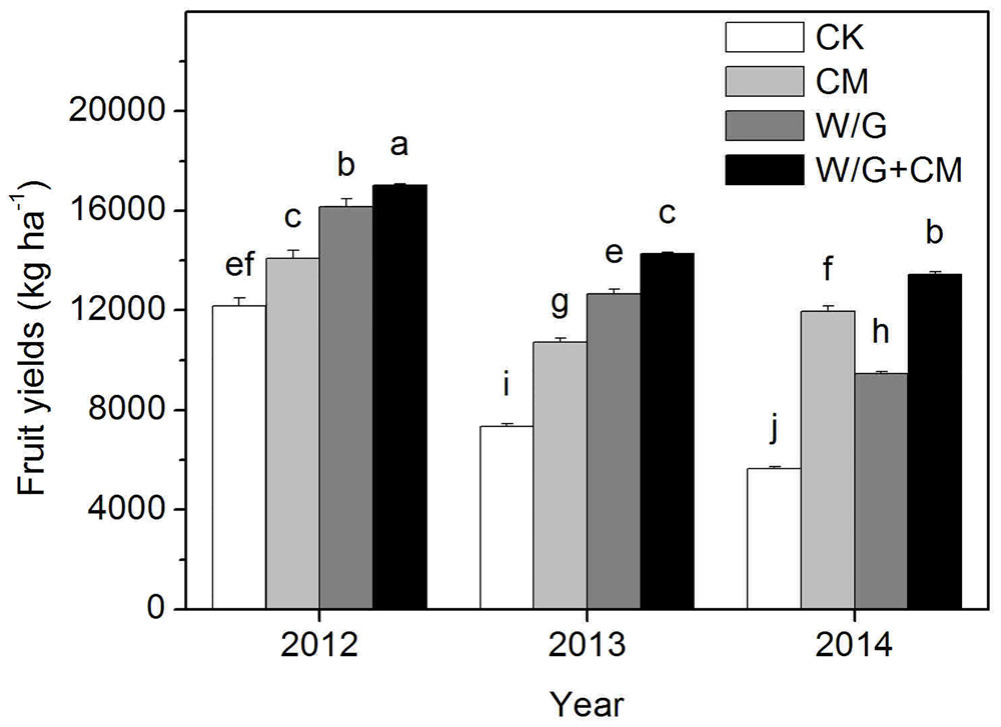
Effect of different treatments on watermelon yields. CK: watermelon continuous cropping; CM: composted cattle manure. W/G: rotation of green garlic with watermelon during the fallow season. W/G+CM: rotation of green garlic with watermelon during the fallow season and composted cattle manure. Different letters on the column indicate a significant difference between the treatments at the *P* < 0.05 level.

Watermelon yield decreased significantly for all the experimental treatments over the three growing seasons (Fig 2). However, the yields for all experimental treatments were significantly higher than that of the control. The yields over the 3-year study period were, in decreasing order, W/G+CM > W/G > CM > CK in the first two years and W/G+CM > CM > W/G > CK in the third year (Fig 2). In the third trial year, cattle manure application and rotation with garlic resulted in watermelon yields of 9,445 (W/G) to 13,441 (W/G+CM) kg ha^−1^, which were 67% and 138% greater than those of the control plots, respectively.

**Fig 3:**
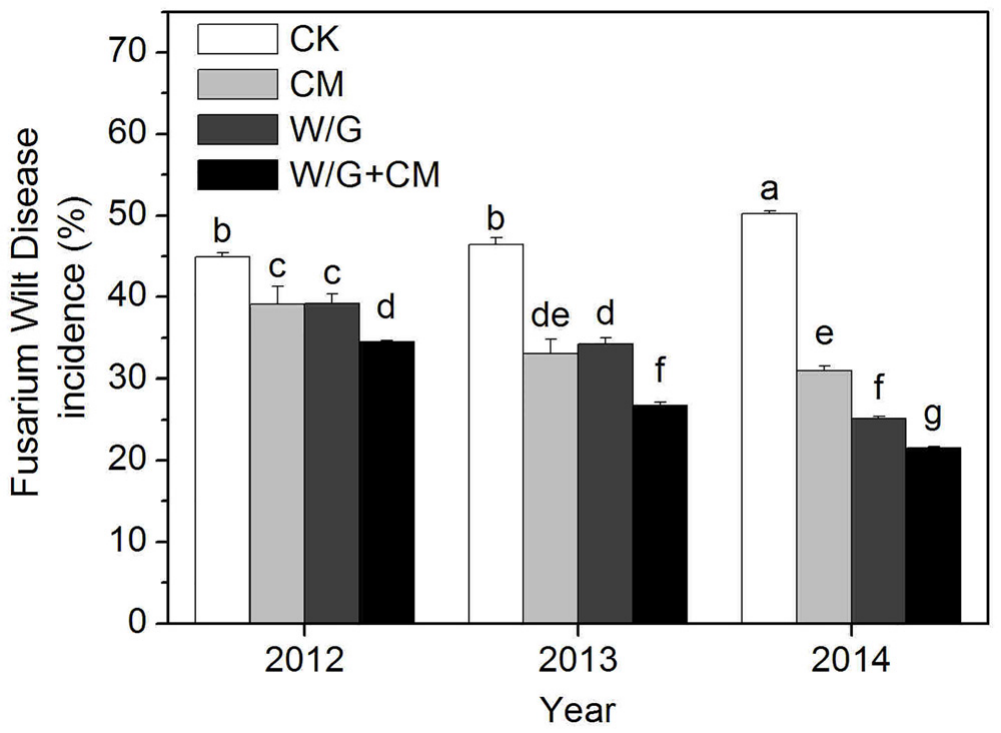
Effect of different treatments on watermelon Fusarium wilt disease incidence. CK: watermelon continuous cropping; CM: composted cattle manure. W/G: rotation of green garlic with watermelon during the fallow season. W/G+CM: rotation of green garlic with watermelon during the fallow season and composted cattle manure. Different letters on the column indicate a significant difference between the treatments at the *P* < 0.05 level.

The effects of each treatment on watermelon *Fusarium* wilt for various numbers of years of continuous cropping are shown in Fig 3. Watermelon disease incidence decreased significantly for the experimental treatments during the entire experimental period, but no difference was detected for the CM and W/G treatments between the first two years. Watermelon disease incidence decreased by 13%, 29%, and 38% for the CM treatment, by 13%, 26%, and 50% for the W/G treatment, and by 23%, 43%, and 57% for the W/G+CM treatment compared with the control in 2012, 2013, and 2014, respectively.

### Effects of cattle manure and garlic rotation on soil biological properties

The populations of soil bacteria, fungi, and actinomycetes have different changes for the experimental treatments and the control (Table 2). The total microbial numbers were significantly higher for the experimental treatments than the control; importantly, rotation with green garlic and the application of cattle manure resulted in the highest microbial populations in all three years. As summarized in Table 2, there was no difference in microbial populations in the control, despite the decreasing numbers of bacteria; as fungi increased, the ratio of bacteria to fungi decreased. The ratio of bacteria to fungi was 18 (CM) to 146 times (W/G+CM) higher for the experimental treatments than the control in the third trial year.

**Table 2:** Soil microbial populations of bacteria, fungi, and actinomyces in different treatments.

Treatment	Micro-biomass(×10^6^ cfu·g^−1^)	Bacteria(×10^6^ cfu·g^−1^)	Fungi(×10^3^ cfu·g^-1^)	Actinomycetes (×10^5^ cfu·g^−1^)	Percentage %			Bacteria/Fungi
					Bacteria	Fungi	Actinomycetes	
2012								
CK	1.01 ± 0.01g	0.78 ± 0.04f	4.08 ± 0.18b	2.37 ± 0.03gh	76.28	0.40	23.32	192.06
CM	1.93 ± 0.03f	1.68 ± 0.06e	3.54 ± 0.35b	2.67 ± 0.04g	86.02	0.18	13.80	470.30
W/G	1.75 ± 0.30f	1.26 ± 0.039ef	2.64 ± 0.17c	2.01 ± 0.01hi	87.73	0.16	12.11	543.18
W/G+CM	2.71 ± 0.18e	2.72 ± 0.097d	1.53 ± 0.42d	3.30 ± 0.12f	87.63	0.06	12.31	1485.58
2013								
CK	1.72 ± 0.00f	1.51 ± 0.01ef	4.10 ± 0.45b	2.03 ± 0.04hi	87.95	0.24	11.81	368.86
CM	5.09 ± 0.08c	4.44 ± 0.13c	2.74 ± 0.11c	6.62 ± 0.14d	86.96	0.05	12.98	1602.60
W/G	3.82 ± 0.19d	3.38 ± 0.29d	1.87 ± 0.03d	4.71 ± 0.10e	87.57	0.05	12.38	1779.52
W/G+CM	8.86 ± 0.11b	8.09 ± 0.39b	1.38 ± 0.09de	6.90 ± 0.11d	92.23	0.02	7.76	5940.70
2014								
CK	0.98 ± 0.02g	0.81 ± 0.03f	4.91 ± 0.33a	1.67 ± 0.06i	82.29	0.50	17.21	163.67
CM	5.49 ± 0.08c	4.45 ± 0.11c	1.50 ± 0.05d	10.49 ± 0.37b	80.89	0.03	19.09	2960.13
W/G	5.50 ± 0.09c	4.57 ± 0.24c	0.74 ± 0.01ef	9.30 ± 0.18c	83.08	0.01	16.91	6161.71
W/G+CM	13.22 ± 0.41a	12.01 ± 0.51a	0.50 ± 0.03f	11.81 ± 0.22a	91.05	0.00	8.95	23948.06

Note: Values are the means ± standard error of three replicates. Different letters indicate a significant difference between the treatments at the *P* < 0.05 level.

**Fig 4:**
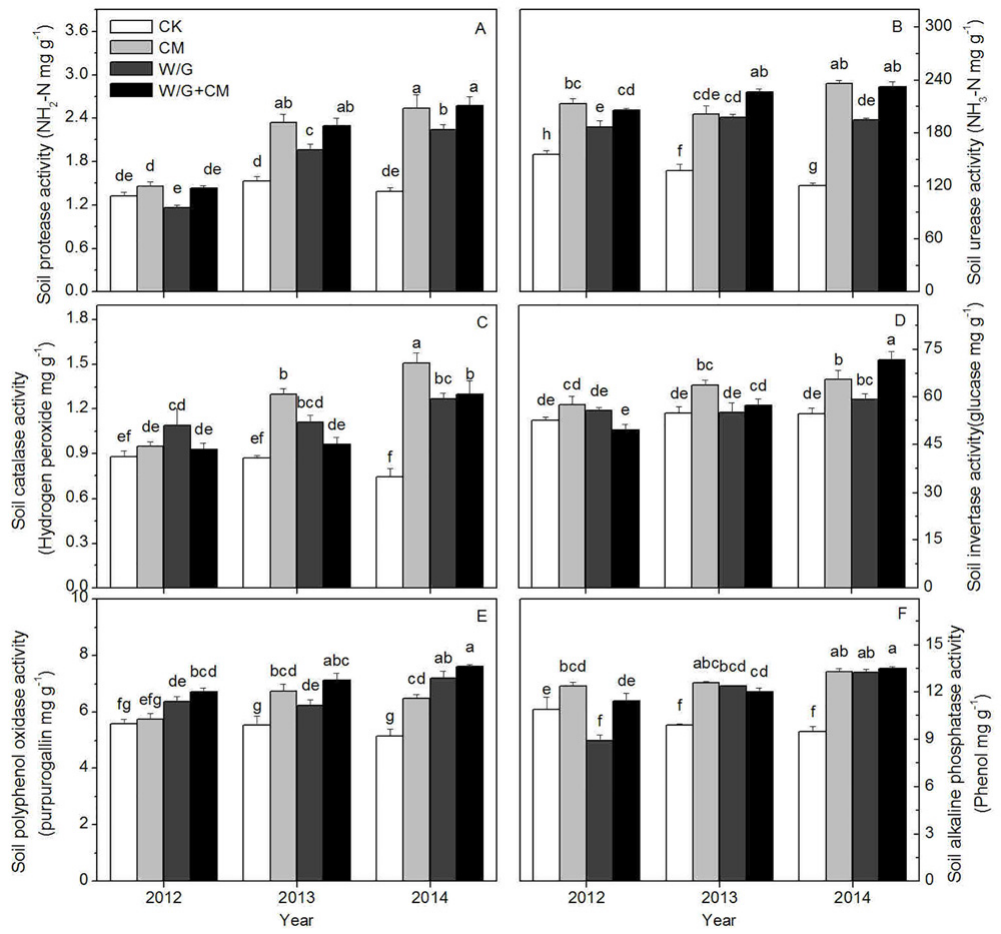
Effects of cattle manure application and garlic rotation on the activities of protease (A), urease (B), catalase (C), invertase (D), polyphenol oxidase (E) and alkaline phosphatase (F) in soil from 2012 to 2014. CK: watermelon continuous cropping; CM: composted cattle manure. W/G: rotation of green garlic with watermelon during the fallow season. W/G+CM: rotation of green garlic with watermelon during the fallow season and composted cattle manure. Different letters on the column indicate a significant difference between the treatments at the *P* < 0.05 level.

After three years, the soil enzyme activities for the experimental treatments increased and were significantly higher than those of the control. The activities of soil protease are shown in Fig 4A. In 2012, the activity values for CM and W/G+CM were higher than those of the control, while there were minimal differences between the other treatments and the control. Rotation with green garlic resulted in the lowest protease activity. In the last two years, the protease activity for each of the treatments increased markedly compared with the first trial year and compared with the control. The application of cattle manure alone and in combination with crop rotation with green garlic resulted in significantly higher protease activities than that of rotation with green garlic alone, but there was no significant difference between the two treatments. Furthermore, there was little change in the protease activity of the control during the three trial years.

As shown in Fig 4B, the overall trend in soil urease activity was similar to that of protease activity. There was a sharp decrease in soil urease activity for all three treatments compared with the control. There were no marked differences in soil urease activity between the treatments, with the exception of W/G. In addition, urease activity of the control declined as the length of continuous cropping increased, and the differences over time were significant.

The soil catalase activity for the experimental treatments changed irregularly over time (Fig 4C). For catalase activity, a continued increase was observed during the three trial years. The W/G treatment showed the highest catalase activity in the first trial year, and there was no significant decrease over the following two years. The catalase activity of the CM treatment exhibited an unusually rapid increase until it reached a maximum in the third year. Although the catalase activity of the W/G+CM treatment did not exhibit significant differences in the first two years, it increased significantly and became the second highest value in the third year.

The soil invertase activity for the experimental treatments was significantly lower than that of the control during the three-year period (Fig 4D); soil invertase activity was highest for the CM treatment, and lowest for the W/G+CM treatment in 2012 and 2013. In 2014, the activity of W/G+CM reached the highest observed value of 71.8 mg g^−1^ glucose.

The activity of soil polyphenol oxidase over the 3-year period was as follows, in decreasing order: W/G+CM > W/G > CM > CK, except in the second year in which CM > W/G (Fig 4E). Activity of soil polyphenol oxidase in the CM treatment initially increased and then decreased, but the decline was non-significant. Although the activity of soil polyphenol oxidase in the control did not differ significantly over time, it decreased each year. There were no differences between the three treatments in alkaline phosphatase activity, except during the first trial year (Fig 4F). For every treatment, alkaline phosphatase activity was much higher in 2014 than in 2012. In contrast, alkaline phosphatase activity in the control decreased remarkably over time.

### Effects of cattle manure and garlic rotation on soil chemical properties

**Fig 5:**
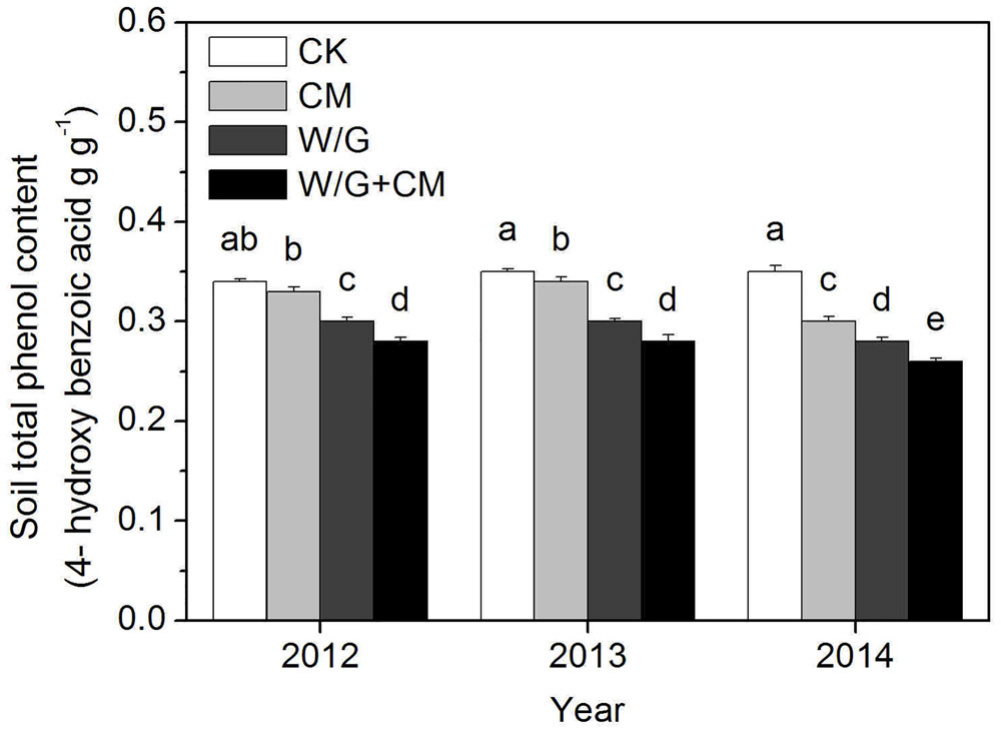
Effects of cattle manure application and garlic rotation on the content of total phenol in soil from 2012 to 2014. CK: watermelon continuous cropping; CM: composted cattle manure. W/G: rotation of green garlic with watermelon during the fallow season. W/G+CM: rotation of green garlic with watermelon during the fallow season and composted cattle manure. Different letters on the column indicate a significant difference between the treatments at the *P* < 0.05 level.

The total phenol content in soil changed regularly and was highest in the control, followed by the CM, W/G, and W/G+CM treatments in every trial year (Fig 5). Along with the increasing length of continuous cropping, total phenol content decreased for all treatments until reaching a minimum and achieved very significant levels in the third year compared with the control. Although there was no significant change in phenol content in the control soil over the three-year period, it tended to increase over time.

**Fig 6:**
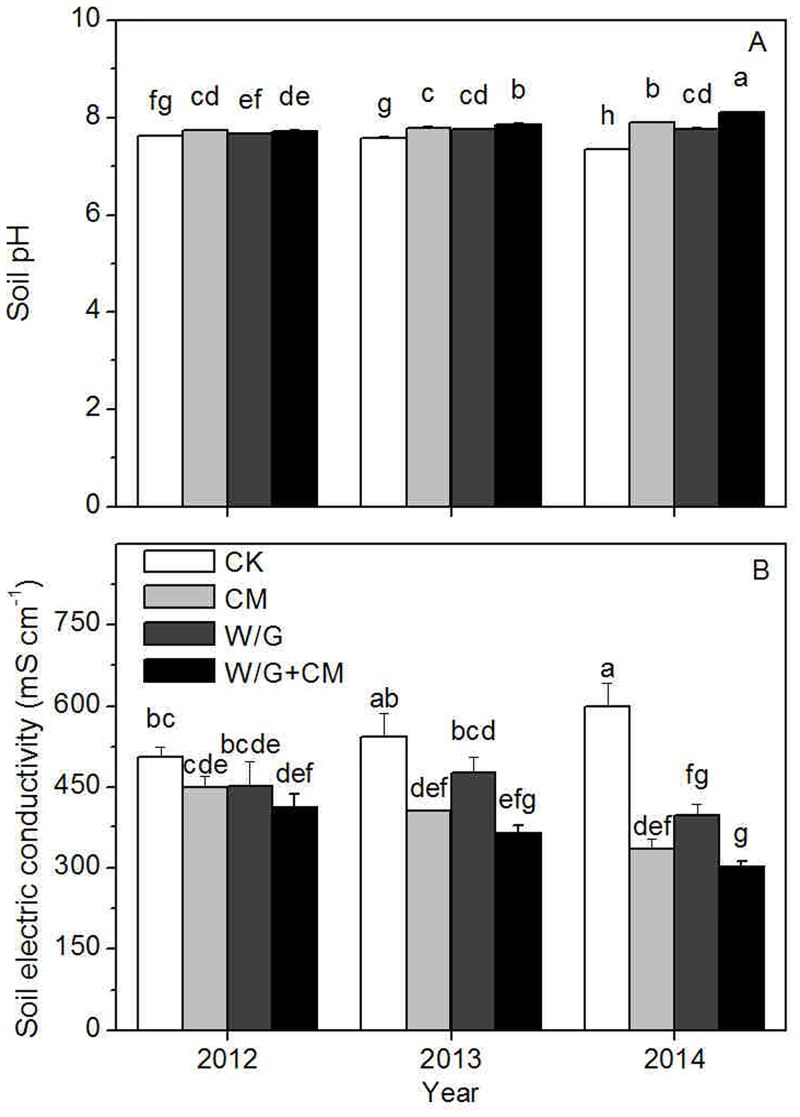
Effects of cattle manure application and garlic rotation on pH (A) and electrical conductivity (B) in soil from 2012 to 2014. CK: watermelon continuous cropping; CM: composted cattle manure. W/G: rotation of green garlic with watermelon during the fallow season. W/G+CM: rotation of green garlic with watermelon during the fallow season and composted cattle manure. Different letters on the column indicate a significant difference between the treatments at the *P* < 0.05 level.

As the length of continuous cropping increased, soil pH in monoculture systems declined gradually and reached a significant level after three years (Fig 6A). After applying cattle manure and rotation with green garlic during the fallow season, soil pH was significantly higher in every continuous cropping year than it was for the monocultures. Except for the W/G treatment in 2012, the pH increased consistently until the end of the study. The differences between soil pH of treatments were significant in the third trial year.

Soil EC increased rapidly in the control in the three continuous cropping years (Fig 6B). It was significantly lower than that of the control for the CM+W/G treatment in 2012, the CM and CM+W/G treatments in 2013, and all treatments in 2014. There were significant differences between some of the treatments; the soil EC was lowest in the W/G+CM treatment, intermediate for the W/G treatment, and highest for the CM treatment.

**Fig 7:**
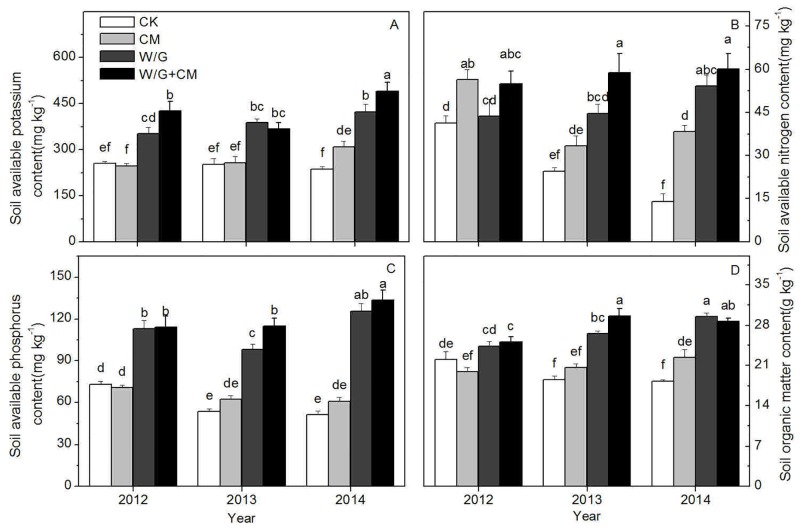
Effects of cattle manure application and garlic rotation on the contents of available potassium (A), available nitrogen (B), available phosphorus (C) and organic matter (D) in soil from 2012 to 2014. CK: watermelon continuous cropping; CM: composted cattle manure. W/G: rotation of green garlic with watermelon during the fallow season. W/G+CM: rotation of green garlic with watermelon during the fallow season and composted cattle manure. Different letters on the column indicate a significant difference among the treatments at the *P* < 0.05 level.

Fig 7A shows that the available K content for the treatments increased continually during the three-year period, but decreased slightly in 2013 for the CM+W/G treatment. The K content for all three treatments was significantly higher in 2014 than in 2012. The available K decreased for the control, but this decrease was not significant. For the experimental treatments, available K increased significantly over time and was significantly higher than that of the control, except for CM in 2012 and 2013.

The available N content was higher for CM+W/G than for the other treatments and the control in most of the sampling years, but reached a peak for the CM treatment in 2012 (Fig 7B). Available N for the W/G and W/G+CM treatments continued to rise, whereas for the CM treatment it decreased initially followed by a slight increase. In 2014, the available N of the three treatments was significantly higher than that of the control. Additionally, the available N for W/G and W/G+CM was almost the same value, but both were significantly higher than that of the CM treatment.

The available P content in soil for the W/G and W/G+CM treatments was significantly higher than that of the control and CM for all time periods (Fig 7C). Although there was no significant difference in available P between CM and the control during the three trial years, it increased slightly over time in the CM treatment, but not in the control. For W/G+CM, the available P content was always higher than that of W/G, but the difference was only significant in 2013.

Organic matter in soil was significantly higher for the three treatments than the control in 2014 (Fig 7D). The results also indicated that there was no significant difference between W/G and W/G+CM in 2013, which were significantly different from the CM treatment and the control in most years. The CM and CK treatments did not exhibit significant differences until the end of the experiment in 2014.

## Discussion

### Effects of cattle manure and garlic rotation on watermelon yield and Fusarium wilt disease

Continuous cropping may have serious negative effects on crop growth and, development and therefore negatively influence yield and quality. Crop rotation [24] and the application of organic manure [25] can improve soil quality, and are more effective means to overcome the obstacles associated with continuous cropping. In this study, we found that garlic rotation and the application of cattle manure significantly increased the production of watermelon via continuous cropping and reduced disease incidence, and that these effects were additive. In addition, to reduce disease incidence in watermelon, garlic rotation was superior to cattle manure. This may be because the root exudates of garlic were inappropriate for *Fusarium* germination or sporulation and thus inhibited *Fusarium* wilt [7]. However, to improve watermelon production, cattle manure was superior to garlic rotation. This may be because cattle manure provides more nutrients, which improves the single fruit weight of watermelon. Furthermore, the influence on yield and the suppression of watermelon *Fusarium* wilt may be related to multiple factors, particularly the alteration of soil biological and chemical properties. In conclusion, we can obtain the highest yield and the lowest disease incidence in the combined treatment.

### Effects of cattle manure and garlic rotation on soil biological properties

There are many kinds of microorganisms in soil (e.g., bacteria, fungi, and actinomycetes) and different microorganisms have different effects on plant growth and development. Generally, most bacteria and actinomycetes found in soil are important for suppressing soil-borne diseases, while fungal species are known to promote soil-borne diseases [26]. Rotation can improve the recovery of soil quality by increasing the levels of viable microorganisms [27], and organic manure amendment has a similar effect [28]. Our data showed that garlic rotation and cattle manure increase the total number of microorganisms. More importantly, they significantly improved bacteria and actinomycetes quantity, reduced the number of fungi, and enhanced the ratio of bacteria to fungi. An increased bacterial population will correspondingly increase microbial activity, and pathogenic bacteria in environments with high microbial activity (diversity) are subjected to higher competition [29]. The ratio of bacteria to fungi was 17-fold higher for the cattle manure treatment than the control. The number of fungi in the soil increased rapidly over time under continuous cropping, and the soil became rich in bacteria after the treatments were applied. Notably, the effect of garlic rotation on fungal inhibition is better than that of cattle manure, perhaps because root exudates of garlic serve as substrates for the microbial community and thus inhibit fungal growth, favoring soil biological quality and soil remediation [30]. Enzyme activities in soil are important markers of soil fertility and are considered to be major factors contributing to overall soil quality and productivity [31]. Previous studies have shown that the extension of the fixed number of years of a crop leads to a reduction in the activity of some soil enzymes, and is an important barrier factor to productivity [32]. However, crop rotation increases soil enzyme activities by 10% to 86% compared with those of continuous cropping [33], similar to the effect of amending soils with organic manure [34]. In our study, the application of garlic rotation and cattle manure independently and combined significantly increased enzyme activity levels in soil (including protease, urease, catalase, invertase, polyphenol oxidase, and alkaline phosphatase activity). For the activity of most enzymes, applying both cattle manure and garlic rotation had a greater effect than any one of the two treatments. It is noteworthy that the enzyme activity levels for treatment with cattle manure were generally higher than for garlic rotation. This might be because increasing cattle manure itself was a kind of enzyme action [30], or because too much residual garlic root and the accumulation of garlic root secretions could produce toxicity, thus inhibiting the enzyme activity in soil [35]. Moreover, the combined treatment was more successful in terms of improving the soil biology than the garlic or manure alone.

Total phenol content in soil, which is one of the main components of root exudates, can indicate improvements in the effect of continuous cropping. The autotoxicity of root exudates, which may contribute to poor soil quality, is one of the most important factors related to the occurrence of continuous monocropping disorders [36]. Previous studies have shown that autotoxic allelopathic potential is minimal for soil treated with farmyard manure [37]. Additionally, 78% of autotoxic substances in root exudates were higher for tobacco continuous cropping than rotation cropping [38]. In our study, the soil phenol content for rotation with garlic was significantly lower than for the cattle manure treatment, likely owing to the higher number of fungi observed for cattle manure than garlic rotation, which inhibited the release of allelochemicals and improved the soil biochemical environment.

### Effects of cattle manure and garlic rotation on soil chemical properties

Soil pH, which not only affects the extent of the decomposition of soil organic matter and nutrient absorption, but also directly affects the reaction rate of soil enzymes involved in biochemical reactions and microbial activity [39], is an important index related to soil properties and crop growth. However, cattle manure amendments can increase soil pH over a short period of time [40]. Higher pH values are observed in soils under rotation than in those under continuous cropping because of the much higher NO_3_^−^ uptake and compensatory exudation of OH^−^ by rotation crops. [41]. In our study, the pH levels for all treatments were higher than that of the control, and the effect of cattle manure was superior to that of rotation, likely due to buffering from bicarbonates and the introduction of elementary cations by cattle manure, such as Ca^2+^ and Mg^2+^ [42]. Soil EC is an indicator of the soil salt content and an important soil property. Excessive salt accumulation in soil not only inhibits soil microbial activity and reduces the rhizosphere nutrient activation, but also causes soil acidification and results in crop nutrition disorders [43]. Accordingly, it is an important indicator of changes in soil quality. The lowest EC values are generally found in the soils under rotation and the application of organic manure has been shown to significantly inhibit the accumulation of salt in the soil [44]. In our study, the EC value was lower for rotation with garlic than for cattle manure application, possibly because garlic absorbed excess salts in the soil. Cattle manure, which had the ability to increase soil pH, may be used to correct soil acidity [45], but can have the negative effect of secondary salinization. Therefore, only the combination of crop rotation with cattle manure can effectively relief the problems of acidification and salinization of soil simultaneously. Soil nutrients are important indicators of soil fertility. Available N, P, K, and organic matter are the main nutrients in the soil. Previous research has demonstrated that N, P, and K use efficiencies, and organic matter in a rotation system are significantly higher than those in a monoculture system [44, 46]. It has also been reported that available N, P, K, and organic matter remain significantly higher in manure-amended soil [47, 48]. Most importantly, organic matter [49], NO_3_^−^-N, and NH_4_^+^-N content [50], which can affect the production and activity of the toxic materials, are negatively correlated with disease incidence. Our study demonstrated that soil nutrient content and enzyme activity showed a similar trend, and rotation with garlic was better than cattle manure to improve soil fertility. In general, the contents of soil nutrients were significantly altered as a result of three years of continuous rotation with garlic, indicating a strong rhizosphere effect that was promoted by the presence of garlic roots. Alternatively, this increase in available nutrients may reflect plant decay after garlic turnover. Therefore, application of cattle manure in combination with garlic rotation significantly increased soil nutrition content and improved soil quality.

## Conclusions

We conclude that the consistent application of cattle manure in combination with garlic rotation resulted in the highest watermelon yield and the lowest disease incidence. This was due to improvements in soil productivity, including enhanced soil biological properties (e.g., microbial quantities and enzyme activity levels), decreased soil chemical characteristics (e.g., total phenol and salt contents), and increased pH and nutrient content. The potential for a decrease in watermelon morbidity was poor if only cattle manure or rotation of garlic was applied. This result indicated that single-repair technology for continuous cropping is not effective. Accordingly, it is necessary to establish a system of multiple repair technologies. Both cattle manure and garlic rotation were shown to be sustainable options for ecological intensification, locally and in other watermelon-growing regions. Further research is needed to examine the potential environmental impact of cattle manure and garlic rotation.
